# Unusual Presentation of Nocardiosis With Pleural Effusion in an Immunocompetent Host

**DOI:** 10.7759/cureus.58686

**Published:** 2024-04-21

**Authors:** Ana Bove, FNU Abdullah, FNU Saveeta, Alexander Urena, Sergio Martinez

**Affiliations:** 1 Medicine, Universidad Iberoamericana (UNIBE), Santo Domingo, DOM; 2 Internal Medicine, Combined Military Hospital, Quetta, PAK; 3 Internal Medicine, People's University of Medical and Health Sciences, Nawabshah, PAK; 4 Medicine, Universidad Tecnológica de Santiago, Santiago de los Caballeros, DOM; 5 Pulmonology, Long Island Jewish Forest Hills, Northwell Health, New York, USA

**Keywords:** right-sided pleural effusion, immunocompetent host, s: pulmonary nocardiosis, loculated pleural effusion, thoracocentesis, empyema, nocardia cyriacigeorgica

## Abstract

Nocardiosis is a disease caused by gram-positive, catalase-positive, rod-shaped bacteria that stain weakly on a Gram stain. It usually affects the lungs and skin but can cause disseminated infections. *Nocardia* has 85 species, ranging from nonpathogenic to pathogenic. *Nocardia* is an opportunistic organism that causes infections in the immunocompromised; however, 7% of the immunocompetent population has suffered from *Nocardia* infection. This case report highlights an unusual occurrence of pulmonary nocardiosis in a 31-year-old woman with a normal immune system. She was initially treated as an outpatient for what appeared to be community-acquired pneumonia. However, her condition deteriorated, ultimately revealing a substantial right pleural effusion with loculation and adjacent compressive atelectasis affecting a significant portion of her right middle and lower lung lobes, as detected by a CT scan followed by pleural fluid analysis which confirmed the infection. By sharing this experience, we aim to contribute to the collective knowledge of medical professionals and improve the accuracy of diagnosis and treatment.

## Introduction

Nocardiosis is an infection caused by *Nocardia*, a bacterium classified as gram-positive, rod-shaped, weakly acid-fast, and weakly staining on a Gram stain, filamentous aerobic actinomycete, that usually is the culprit of opportunistic infection in immunocompromised patients. The aerobic characteristic of this pathogen leads to its predominance in pulmonary infections, but it can also be disseminated [1]. Although nocardial infections often cause opportunistic infections in the immunocompromised host, at least one-third of the infections can occur in patients without a definable risk factor [2]. These risk factors are associated with decreased immune response, such as cancer, diabetes, chemotherapy, human immunodeficiency virus (HIV), autoimmune disease, etc.

*Nocardia* species are worldwide natural dwellers of the soil. Pulmonary nocardiosis is usually caused by the direct inhalation of *Nocardia* spp. from contaminated soil, and person-to-person transmission is uncommon [3]. *Nocardiaceae* is a large family of species; however, the isolated bacterium in this case report was *Nocardia cyriacigeorgica*. This particular species was first discovered in 2001, and multiple strains of *Nocardia cyriacigeorgica *have been recovered since as the causative agent of human infection in countries all around the world. Most cases have occurred in the context of HIV-related or iatrogenic immune suppression [4]. This is a case report of a 31-year-old female, immunocompetent, with a prominent pleural effusion caused by *Nocardia cyriacigeorgica*, without definable predisposing risk factors, a striking feature of this case.

## Case presentation

A 31-year-old female presented to the outpatient clinic with a seven-day history of fever, chills, dry cough, and pleuritic and back pain, who works at a supermarket, packing fresh products. She denied recent travel, smoking, and any significant medical history, including diabetes, hypertension, asthma, and contraceptive use. Initial evaluation revealed a temperature of 38.1°C, a SpO2 of 93% on room air, a respiratory rate of 20 breaths per minute, a heart rate of 106 beats per minute, and a blood pressure of 120/80 mm Hg. The physical examination revealed bibasilar lung crackles on auscultation.

**Figure 1:**
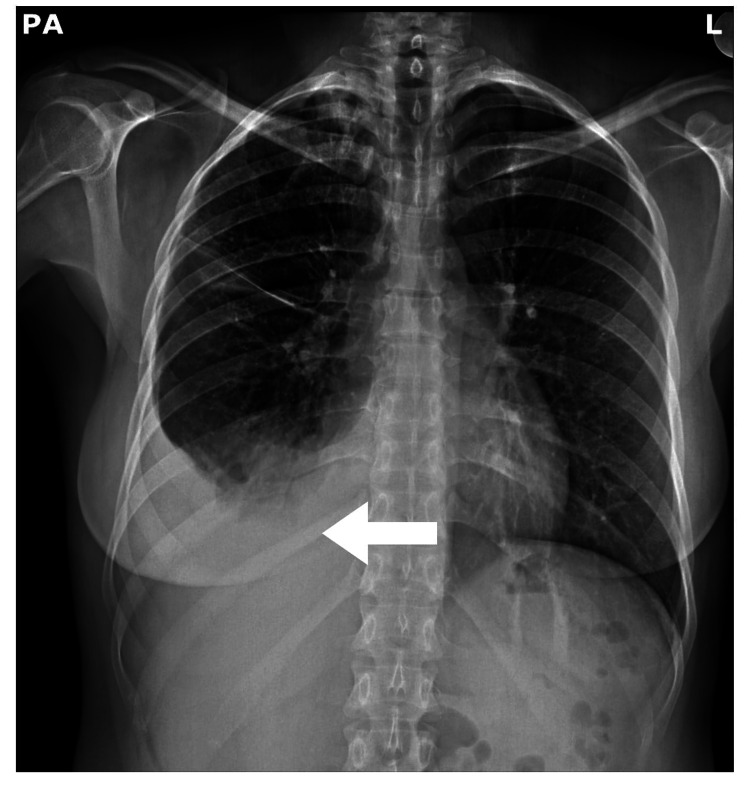
CXR PA Plain chest radiograph PA view. Arrow showing right lower lobe consolidation and right mild pleural effusion CXR: chest X-ray; PA: posteroanterior

A few investigations were ordered, including a complete blood count (CBC), a complete metabolic panel (CMP), both a coronavirus disease 2019 (COVID-19) rapid test and a polymerase chain reaction (PCR) test, and a chest X-ray (CXR). The CBC showed a hemoglobin level of 9.0 g/dl, a mean corpuscular volume (MCV) of 88 fl, a white blood cell count of 10.5 M/uL, and a platelet count of 367K/uL. The CMP was unremarkable. The COVID-19 test came back negative, and inflammatory markers indicated an erythrocyte sedimentation rate (ESR) of 100. The CXR (Figure 1) showed right lower lobe consolidation, and the patient was started on oral antibiotics for community-acquired pneumonia.

Five days later, she presented to the emergency department with worsened shortness of breath and an oxygen saturation of 88% on room air. A subsequent CXR revealed a large right pleural effusion and patchy right upper lobe infiltrate of either infectious or malignant etiology. A repeat COVID-19 test was negative. A chest CT scan showed a large, partially loculated right pleural effusion with adjacent areas of compressive atelectasis involving significant portions of the right middle and lower lobes and an indeterminate patchy opacity in the right upper lobe. The patient was moved to the ICU and was provided the required respiratory support.

Comprehensive testing for *Mycoplasma*, *Legionella*, *Streptococcus pneumoniae*, acid-fast bacilli (AFB), sputum culture, blood cultures (BCx), and respiratory viral panel (RVP) all returned negative results. However, the patient tested positive for *Mycoplasma* IgM. Thoracocentesis was performed, and a chest tube was inserted, yielding an output of 700 cc in 24 hours. A pleural fluid sample was collected for analysis to rule out the possible differentials and establish a diagnostic cause; findings are mentioned in Table 1. Intravenous broad-spectrum empiric antibiotics twice a day were initiated immediately for suspected parapneumonic effusion or empyema, as well as RIPE (rifamycin (like rifampin), isoniazid, pyrazinamide, and ethambutol) therapy for suspected pleural tuberculosis (TB).

**Table 1:** Pleural fluid analysis

Parameters	Results	Reference range
Color	Slightly cloudy	Clear
pH	8.2	7.60-7.64
Lactate dehydrogenase	329	<50% plasma concentration
Total protein	5.5	1-2 g/dl
Albumin	2.9	3.5-5.5 (g/dL)
Glucose	85	70-100 mg/dl
Adenosine deaminase, PI	40	0-30 (U/L)

Additional TB tests conducted, including a negative QuantiFERON, TB complex PCR, and sputum culture for AFB, came back negative. Further investigations for possible immunodeficiency, including HIV testing, serum quantitative immunoglobulins, and a viral hepatitis panel, were negative, ruling out immunodeficiency. Tumor markers such as CA 19-9 and carcinoembryonic antigen were also negative. The pleural biopsy revealed numerous necrotizing granulomas with multinucleated giant cells and a mixture of acute and predominantly chronic inflammation.

The patient improved on antibiotics and symptomatic treatment. After 10 days of clinical improvement with 99% oxygen saturation on room air, the patient was discharged, RIPE therapy was discontinued as TB results were negative, and the patient was requested to follow up as an outpatient. Two weeks later, the blood cultures came back negative. The pleural fluid culture results isolated *Nocardia cyriacigeorgica, *and the patient was informed of the diagnosis during follow-up as an outpatient. Treatment with trimethoprim/sulfamethoxazole (TMP/SMX) was initiated and continued for six months. The patient reported improved symptoms, including the absence of cough, chest pain, and fever, while continuing treatment with TMP/SMX on subsequent outpatient follow-up visits.

## Discussion

According to the Centers for Disease Control and Prevention (CDC), the incidence of nocardiosis is 500-1000 cases per year in the United States [5]. Nocardiosis most commonly infects the lung, but if left untreated, the infection can easily spread to other body compartments, including the brain, which can result in a fatal outcome; given that, up to 44% of all patients with brain or spinal cord infection die. The brain is the most common site of disseminated infection [6].

As Conville and Witebsky described, *Nocardia cyriacigeorgica* is coincident with strains classified as having a type VI drug pattern. In addition to sulfonamide susceptibility, type VI strains are mainly susceptible to broad-spectrum cephalosporins, amikacin, imipenem, and linezolid yet resistant to penicillin, clarithromycin, and ciprofloxacin [7].

Historically, infections with *Nocardia* were associated with exceedingly high mortality rates. In view of the previously mentioned drug pattern sensitivity, the treatment with sulfonamides has markedly decreased mortality, and TMP/SMX is the mainstay treatment for nocardiosis in the United States. However, combination treatment is preferred, especially in severe, disseminated infections [8]. 

TMP/SMX is the preferred treatment in pulmonary nocardiosis. It has the additional advantage of exceptional penetration into most tissue compartments including the lungs, pericardium, and mediastinum and high serum concentrations after oral administration. Certain *Nocardia* isolates may be susceptible to sulfonamides or TMP/SMX, and a favorable response to treatment may be established in 90% or more of cases if the infection is limited to pleuropneumonia. For the majority of patients with nocardiosis, clinical improvement is expected within 7-10 days after the initiation of empiric therapy with sulfonamides (with or without trimethoprim) [9].

The significance of this case report is highlighted by the intricate challenge in diagnosing pulmonary nocardiosis in an immunocompetent individual. This intricate diagnostic process is further compounded by the potential gravity of the situation: untreated cases could culminate in fatal outcomes for the affected patient. 

## Conclusions

Indeed, nocardiosis, caused by the *Nocardia* species, has gained recognition as an emerging disease in the United States. The evolving nature of infectious diseases, such as in the case mentioned above, requires vigilant attention from health professionals to broaden their diagnostic considerations. Nocardiosis, traditionally associated with immunocompromised individuals, has demonstrated the ability to affect immunocompetent individuals as well, as evidenced by the case at hand. This shift in presentation emphasizes the necessity for healthcare practitioners to expand their diagnostic horizons, considering even the less conventional causative agents while treating patients.

Nocardiosis is an infection that, if disseminated, has a high mortality rate; hence, it is important to diagnose it immediately, especially when it is pulmonary-associated since half of the disseminated cases are secondary to pulmonary involvement. Consequently, early recognition and management of pulmonary nocardiosis, even within immunocompetent patients, are pivotal in slowing the progression of the infection and preventing its potentially severe consequences.

## References

[1] Martínez Tomás R, Menéndez Villanueva R, Reyes Calzada S, Santos Durantez M, Vallés Tarazona JM, Modesto Alapont M, Gobernado Serrano M (2007). Pulmonary nocardiosis: risk factors and outcomes. Respirology.

[2] Beaman BL, Burnside J, Edwards B, Causey W (1976). Nocardial infections in the United States, 1972-1974. J Infect Dis.

[3] Menéndez R, Cordero PJ, Santos M, Gobernado M, Marco V (1997). Pulmonary infection with Nocardia species: a report of 10 cases and review. Eur Respir J.

[4] Schlaberg R, Huard RC, Della-Latta P (2008). Nocardia cyriacigeorgica, an emerging pathogen in the United States. J Clin Microbiol.

[5] Rathish B, Zito PM (2023). Nocardia. StatPearls [Internet].

[6] Rathish B, Zito PM (2023). Nocardiosis: Transmission. StatPearls.

[7] Conville PS, Witebsky FG (2007). Organisms designated as Nocardia asteroides drug pattern type VI are members of the species Nocardia cyriacigeorgica. J Clin Microbiol.

[8] Brown-Elliott BA, Brown JM, Conville PS, Wallace RJ Jr (2006). Clinical and laboratory features of the Nocardia spp. based on current molecular taxonomy. Clin Microbiol Rev.

[9] Bell M, McNeil MM, Brown JM (2023). Nocardia species (nocardiosis). J Infect Dis.

